# Advances in the role of specialized pro-resolving mediators in periodontitis

**DOI:** 10.3389/fimmu.2026.1883801

**Published:** 2026-09-04

**Authors:** Huiting Hu, Hongxing Chu, Zhenzhen Wu, Zekun Gan, Yong Yang

**Affiliations:** 1 Department of Oral Medicine, Stomatological Hospital, School of Stomatology, Southern Medical University, Guangzhou, China; 2 Department of Periodontics and Implantology, Stomatological Hospital, School of Stomatology, Southern Medical University, Guangzhou, China

**Keywords:** host-modulatory therapy, inflammation resolution, lipoxins, maresins, periodontal regeneration, periodontitis, resolvins, specialized pro-resolving mediators

## Abstract

Periodontitis is a chronic inflammatory disease driven by dysregulated host–microbial interactions and failure of endogenous inflammation resolution, leading to progressive destruction of periodontal tissues. Increasing evidence indicates that inflammation resolution is an active, tightly regulated biological program governed by specialized pro-resolving mediators (SPMs), including resolvins, lipoxins, protectins, and maresins. Unlike conventional anti-inflammatory agents, SPMs do not suppress host defense but instead actively terminate inflammation and promote tissue regeneration. Mechanistically, SPMs signal through specific G protein-coupled receptors to inhibit neutrophil recruitment, enhance macrophage efferocytosis, and promote M2 macrophage polarization. They also suppress pro-inflammatory signaling pathways such as NF-κB and NLRP3 inflammasome activation, while restoring immune balance by modulating Treg/Th17 responses. Importantly, SPMs regulate periodontal bone homeostasis by correcting the RANKL/OPG imbalance and supporting osteogenic differentiation of periodontal ligament cells. This review summarizes the biosynthesis, receptor-mediated signaling, and functional roles of SPMs in periodontitis, highlighting their may connect inflammation resolution and tissue regeneration.

## 1 Introduction

Periodontitis is a chronic inflammatory disease characterized by the progressive destruction of periodontal supporting tissues, including the gingiva and alveolar bone (1, 2). Severe periodontal disease affects approximately 1 billion people worldwide, underscoring its substantial public-health burden (3). Beyond tooth loss and impaired quality of life, periodontitis is associated with diabetes mellitus, cardiovascular disease, and rheumatoid arthritis through shared risk factors, episodic bacteremia, and systemic inflammatory pathways; these associations should not be interpreted as proof of causality in every clinical setting (4–6). It is increasingly viewed not merely as a state of excessive inflammation, but as a complex pathology of failed inflammation resolution (7). Inflammation resolution is an active biological program governed by specialized pro-resolving mediators (SPMs) (8). Unlike traditional anti-inflammatory drugs, SPMs are endogenous lipid mediators that actively terminate inflammation and promote tissue regeneration without broadly compromising host defense (9, 10). Conventional periodontal therapy, including oral-hygiene measures, supra- and subgingival instrumentation, and surgery when indicated, reduces dysbiotic biofilm and inflammatory burden, but previously lost attachment and alveolar bone are not predictably restored, and resolution may remain impaired in susceptible patients with persistent risk factors or dysregulated host responses (2, 5, 11). SPMs have therefore merged as potential adjunctive host-modulatory agents rather than replacements for mechanical infection control.

At the cellular level, SPMs limit neutrophil (polymorphonuclear leukocyte, PMN) recruitment, promote neutrophil apoptosis, and enhance macrophage efferocytosis (12, 13). They also favor a pro-resolving, M2-like macrophage phenotype. Molecularly, SPMs can suppress inflammatory TLR4/MyD88/NF-κB and NLRP3 signaling, reduce pro-inflammatory cytokines and reactive oxygen species, and regulate the Treg/Th17 balance. Furthermore, they may facilitate periodontal regeneration by correcting the RANKL/OPG imbalance to restrain osteoclastogenesis while supporting osteogenic differentiation of periodontal ligament stem cells (14). Several comprehensive reviews have summarized the general biology and periodontal applications of SPMs (7–11, 15–17). The added value of the present review is an integrated comparison of precursor-enzyme pathways, ligand-receptor assignments, intracellular signaling, periodontal evidence tiers, and practical translational barriers. Accordingly, this review covers SPM biosynthesis and lipid mediator class switching, receptor biology, intracellular signaling pathways, *in vitro* and animal evidence, the limited human evidence, advanced delivery, precision periodontology, and scalable production.

## 2 Initiation and resolution of inflammation

Inflammation constitutes a fundamental host-defense response to microbial invasion or tissue injury, characterized by vasodilation, increased vascular permeability, leukocyte recruitment, and edema (18, 19). Upon microbial challenge, pathogen-associated molecular patterns (PAMPs) engage pattern-recognition receptors (PRRs) on resident cells, triggering synthesis of pro-inflammatory mediators—including TNF-α, IL-1β, IL-6, chemokines, and prostaglandins—and activating intracellular signaling cascades such as NF-κB and the NLRP3 inflammasome, which amplify and sustain the inflammatory response (4, 20–22). Under physiological conditions, inflammation is self-limiting; its resolution represents an active, SPM-mediated “programmed” process essential for tissue homeostasis restoration (12, 23).

Within the periodontal microenvironment, disease progression is spatially and temporally dynamic. In health, junctional epithelial cells, fibroblasts, resident macrophages, and a controlled neutrophil surveillance network maintain barrier integrity. Plaque accumulation produces gingivitis, with vascular activation and increased neutrophil recruitment that is initially reversible. In susceptible hosts, transition to periodontitis is accompanied by persistent neutrophil dysfunction, influx of monocytes and dendritic cells, expansion of Th1/Th17 and B-cell/plasma-cell populations, fibroblast and epithelial activation, increased RANKL expression, and osteoclast-mediated alveolar bone loss. Advanced lesions therefore contain overlapping destructive and reparative cell states rather than a single linear immune phase, and stage/grade, smoking, diabetes, and microbial composition modify this trajectory (2, 20, 24, 25).

Innate and stromal mechanisms form the core of the resolution program. SPM generation, cessation of further neutrophil recruitment, macrophage efferocytosis, and clearance of inflammatory debris help terminate feed-forward NF-κB/NLRP3 activity and create conditions for repair (26–34). Adaptive immunity is considered here as a downstream layer rather than the principal focus: available evidence indicates that SPMs may restrain excessive Th1/Th17 polarization, support regulatory T-cell responses, and influence B-cell function (2, 10, 11, 30, 35–37). SPMs counter-regulate PAMP–PRR signaling by suppressing NF-κB activation, limiting NLRP3 inflammasome assembly, and reducing IL-1β maturation (30–32), while enhancing efferocytosis to eliminate inflammasome-activating cellular debris and dampen inflammatory amplification loops (15, 33, 34). Failure of this self-limiting process predisposes to chronic inflammatory pathologies, including periodontitis, cardiovascular and neurodegenerative diseases, and metabolic syndromes (38, 39).

In periodontal tissues, homeostasis is governed by dynamic interactions among dental plaque biofilms, host-derived inflammatory and pro-resolving mediators, and local/systemic risk factors (5, 40). Periodontitis results from an imbalance among these factors and manifests as chronic inflammatory destruction of periodontal supporting tissues. Key periodontal pathogens activate multiple host-defense mechanisms. For example, lipopolysaccharide (LPS) from *Porphyromonas gingivalis* (*P. gingivalis*) stimulates inflammatory mediator and reactive oxygen species production, contributing to matrix degradation and oxidative stress (24, 41). Neutrophils and monocytes/macrophages migrate to inflamed sites to phagocytose bacteria (42, 43). During resolution, these leukocytes produce SPMs that support microbial clearance, limit continued inflammatory recruitment, and create a tissue environment permissive for epithelial, connective-tissue, and bone repair (11, 12) (**Supplementary Figure 1**).

## 3 Specialized pro-resolving mediators in periodontitis

SPMs are lipid-derived bioactive molecules generated through stereoselective enzymatic metabolism of omega-6 and omega-3 polyunsaturated fatty acids by cyclooxygenases (COXs), lipoxygenases (LOXs), cytochrome P450 (CYP) enzymes, and transcellular pathways (9, 44, 45). Four precursor-enzyme axes are particularly relevant. Arachidonic acid (AA) yields lipoxins through sequential 15-LO/5-LO or leukocyte 5-LO/platelet 12-LO reactions; aspirin-acetylated COX-2 or CYP generates 15R-HETE for aspirin-triggered lipoxins. Eicosapentaenoic acid (EPA) yields E-series resolvins through 18-HEPE formation followed principally by 5-LO–dependent reactions. Docosahexaenoic acid (DHA) yields D-series resolvins and protectins through 15-LO/5-LO–related pathways and maresins th4rough macrophage 12-LO–initiated 13S, 14S-epoxide chemistry. n-3 docosapentaenoic acid (n-3 DPA) gives rise to T-series resolvins and n-3 DPA-derived resolvins, protectins, and maresins through pathway-specific COX-2/LOX reactions (44–47). Receptor assignments are ligand-specific rather than uniform across an SPM family: examples with the strongest evidence include LXA4–ALX/FPR2, RvD1–ALX/FPR2 and GPR32, RvD2–GPR18, RvE1–ERV1/ChemR23 and BLT1, PD1/NPD1–GPR37, and MaR1–LGR6 (48–54). Several SPM receptors remain unresolved, which is explicitly indicated in **Table 1**. Through these receptors, SPMs limit excessive PMN recruitment, enhance macrophage phagocytosis and efferocytosis, favor pro-resolving macrophage states, modulate NF-κB, ERK/MAPK, PI3K/Akt, Nrf2, and inflammasome signaling in a cell- and ligand-dependent manner, and support microbial clearance and tissue repair (16, 94). Unlike non-selective inhibition of prostaglandin synthesis, SPM pharmacology aims to activate endogenous resolution programs while preserving antimicrobial defense (95).

**Table 1:** Comparative summary of major specialized pro-resolving mediator families relevant to periodontitis.

SPM family	Precursor and principal enzymes	Best-supported receptor(s)	Major actions/signaling	Periodontal evidence and status
Lipoxins (LXA4, LXB4, AT-LXs)	AA; 15-LO/5-LO or leukocyte 5-LO/platelet 12-LO; aspirin-COX-2/CYP → 15R-HETE	LXA4/AT-LXA4: ALX/FPR2	Limits PMN recruitment; promotes efferocytosis; modulates NF-κB, ERK/MAPK, Nrf2	Periodontal cell and animal studies; one phase-1 BLXA4 trial in gingival inflammation (55–63)
E-series resolvins (RvE1–RvE4)	EPA; 18-HEPE formation followed mainly by 5-LO; RvE4 uses a distinct 15-LO/5-LO route	RvE1: ERV1/ChemR23 and BLT1; RvE2: BLT1; others unresolved	Restrains LTB4/PMN signaling; improves phagocytosis and microbial clearance	Multiple periodontal preclinical studies; no approved periodontal formulation (64–67)
D-series resolvins (RvD1–RvD6)	DHA; 15-LO then 5-LO; aspirin-COX-2/CYP can generate 17R epimers	RvD1: ALX/FPR2, GPR32; RvD2: GPR18; evidence for RvD3/RvD5: GPR32	Modulates PI3K/Akt and ERK/MAPK; suppresses inflammatory NF-κB; supports efferocytosis	Periodontal cell and animal studies; human efficacy unestablished (68–76)
Protectins (PD1/NPD1, PDX)	DHA; mainly 15-LO–initiated pathways	PD1/NPD1: GPR37 on macrophages; PDX receptor assignment unresolved	Limits leukocyte trafficking; GPR37-dependent macrophage phagocytosis/efferocytosis	Periodontal-specific evidence is sparse; most mechanistic evidence is non-periodontal (54, 77–84)
Maresins (MaR1, MaR2)	DHA; macrophage 12-LO → 13S, 14S-epoxide; enzymatic hydrolysis to MaR1/MaR2	MaR1: LGR6; receptors for MaR2 and several congeners unresolved	Promotes efferocytosis and pro-resolving/M2-like states; can engage ERK/CREB and Nrf2	Limited periodontal cell/rodent wound evidence; much evidence is extrapolated (50, 85–92)
n-3 DPA-derived mediators/T-series resolvins	n-3 DPA; COX-2/5-LO or LOX-specific pathways depending on mediator	Largely unresolved	Limits PMN infiltration and supports bacterial clearance in non-periodontal models	Periodontal roles remain insufficiently characterized (46, 47, 81, 93)

Receptor assignments reflect the best-supported pairings available at the time of revision; several SPM family members still lack definitively established receptors. AT, aspirin-triggered; AA, arachidonic acid; EPA, eicosapentaenoic acid; DHA, docosahexaenoic acid; n-3 DPA, n-3 docosapentaenoic acid.

### 3.1 Resolvins

**Figure 1:**
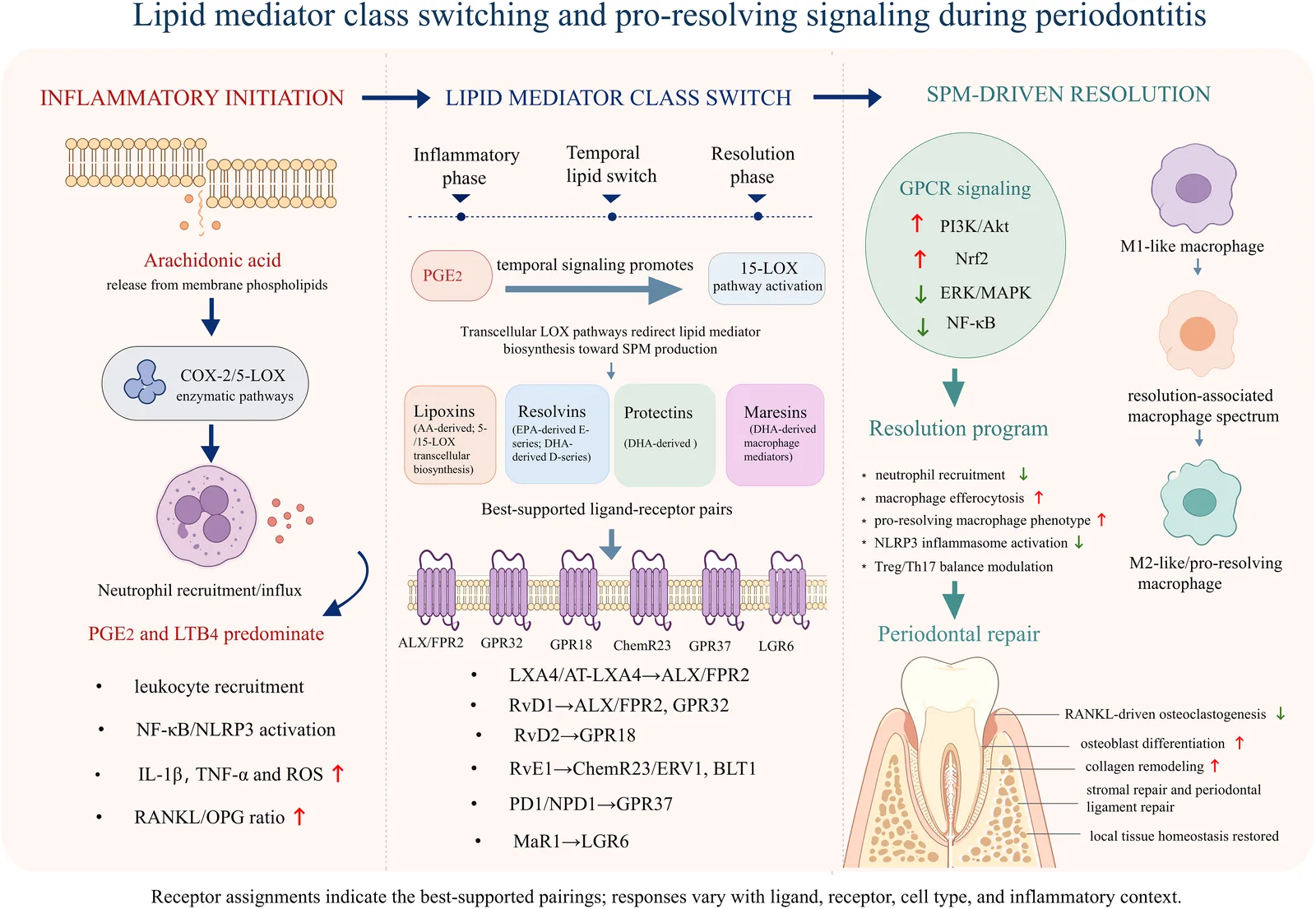
Integrated model of lipid mediator class switching, major SPM-receptor pairings, context-dependent intracellular signaling, and periodontal cellular outcomes. Inflammatory initiation releases arachidonic acid from membrane phospholipids, generating prostaglandin E2 and leukotriene B4 through cyclooxygenase and lipoxygenase pathways and promoting neutrophil recruitment, cytokine production, inflammasome activation, and osteoclastogenesis. Temporal prostaglandin E2 signaling activates 15-lipoxygenase and redirects lipid biosynthesis toward specialized pro-resolving mediators, including lipoxins, resolvins, protectins, and maresins. These mediators engage G-protein-coupled receptors to promote macrophage efferocytosis and pro-resolving polarization while limiting neutrophil influx, NF-κB/NLRP3 signaling, and RANKL-dependent bone resorption. The resulting resolution program restores homeostasis and supports osteoblast differentiation, collagen remodeling, periodontal ligament repair, and tissue regeneration.

Resolvins are enzymatically derived from omega-3 polyunsaturated fatty acids and include D-series (RvD), E-series (RvE), and T-series (RvT) subfamilies (96, 97). RvD1–RvD6 are formed from DHA predominantly through 15-LO–initiated and 5-LO–dependent pathways; aspirin-acetylated COX-2 or CYP can generate 17R epimers rather than serving as the universal route for native RvDs. EPA is converted to 18-HEPE by aspirin-acetylated COX-2 or CYP-related pathways and then principally by 5-LO to RvE1 and RvE2, whereas RvE3 and RvE4 use distinct enzymatic routes (44, 98). T-series resolvins arise from n-3 DPA through endothelial COX-2/leukocyte 5-LO cooperation and should be distinguished from the separate n-3 DPA-derived D-series-like mediators (46, 47). Receptor-ligand mapping clarifies their functional diversity: RvD1 activates ALX/FPR2 and GPR32; RvD2 activates GPR18; RvE1 activates ERV1/ChemR23 and also interacts with BLT1; RvE2 interacts with BLT1; and evidence supports GPR32 engagement by RvD3 and RvD5, while receptors for several other resolvins remain unresolved (36, 48, 49, 51–53). ERV1/ChemR23, GPR32, and GPR18 signaling promotes phagocyte clearance and pro-resolving programs, whereas RvE1 competition/partial agonism at BLT1 counter-regulates LTB4-driven PMN recruitment. These pathways can engage PI3K/Akt and ERK/CREB while limiting inflammatory NF-κB/MAPK output in a cell- and context-dependent manner. Periodontally, RvDs reduce the RANKL/OPG ratio and alveolar bone loss (69), enhance periodontal ligament cell migration and proliferation (70, 71), support macrophage bacterial clearance and Treg functions (72, 73), and counteract IL-17-mediated suppression of Del-1 (76). RvE1 regulates periodontal ligament cell responses and inflammatory cytokines (70, 99), restores impaired macrophage phagocytosis ex vivo in leukocytes from localized aggressive periodontitis (99), and reverses inflammation, dysbiosis, and bone loss in animal models (65–67, 74). RvTs reduce PMN infiltration and support bacterial clearance in non-periodontal models (93), but their periodontal roles and those of several receptor-unresolved resolvins remain incompletely characterized (**Figure 1**).

### 3.2 Lipoxins

Lipoxins (LXs) are arachidonic acid (AA)-derived trihydroxytetraenes; major members include LXA4, LXB4, and aspirin-triggered lipoxins (AT-LXs) (100, 101). They are generated through three principal routes. First, AA is sequentially oxygenated by 15-LO and 5-LO in mucosal and leukocyte-rich tissues. Second, neutrophil 5-LO products can be transferred to platelets and converted by platelet 12-LO in a transcellular route. Third, aspirin-acetylated COX-2 or CYP generates 15R-HETE, which is converted by leukocyte 5-LO into 15-epi-LXA4 and 15-epi-LXB4 (101–107). LXA4 and AT-LXA4 signal mainly through ALX/FPR2 and provide endogenous stop signals that limit PMN adhesion, transendothelial migration, and chemotaxis while supporting efferocytosis and repair (48, 55, 56). *In vivo* periodontal evidence includes reduced alveolar bone loss and local inflammation in 15-LO–overexpressing transgenic rabbits after ligature and P. gingivalis challenge, as well as protection by local stable lipoxin analogues in non-transgenic periodontitis models (57–59).

*In vitro* periodontal evidence shows that LXA4 suppresses LPS-induced pro-inflammatory mediator expression in periodontal ligament cells by inhibiting TLR4/MyD88/NF-κB signaling and can partially reverse macrophage-mediated inhibition of these cells under inflammatory conditions (56, 60). LXA4 also suppresses P. gingivalis LPS-induced inflammasome-associated activation in macrophages (62). As non-periodontal mechanistic support, peritonitis experiments demonstrated that LXA4, 15-epi-LXB4, and stable LXA4 analogues enhance macrophage uptake of apoptotic PMNs *in vivo* (61). Organizing the evidence by model therefore shows that periodontal cell and animal studies support anti-inflammatory and regenerative effects, whereas some efferocytosis mechanisms are derived from other inflammatory systems and require periodontal confirmation.

### 3.3 Protectins

Docosahexaenoic acid (DHA) is converted by 15-LO–initiated pathways in PMNs, macrophages, eosinophils, and other cells to 17-HDHA and then protectin D1 (PD1); in neural tissues PD1 is termed neuroprotectin D1 (NPD1) (77–79). Aspirin-acetylated COX-2 can support formation of aspirin-triggered protectin epimers, and DHA can also give rise to the double-lipoxygenation isomer protectin DX (PDX) (80). n-3 DPA is a substrate for related PD1n-3 DPA and PD2n-3 DPA mediators (81). PD1/NPD1 limits neutrophil chemotaxis and tissue accumulation by counter-regulating leukocyte trafficking signals; in parallel, NPD1/PD1 engagement of macrophage GPR37 induces calcium-dependent phagocytosis and efferocytosis, thereby indirectly shortening neutrophil persistence at inflammatory sites (54, 83). PDX reduces neutrophil infiltration in non-periodontal models through suppression of TNF-α/MIP-2/MMP signaling (82). PD1 and PDX also reduce pro-inflammatory cytokine release and inflammatory pain in several experimental systems (83, 84). Importantly, these mechanistic findings derive largely from peritonitis, pulmonary, vascular, neural, or postoperative-pain models. Direct periodontal data for PD1, PDX, aspirin-triggered PD1, and n-3 DPA protectins remain sparse; periodontal efficacy and receptor biology should therefore be considered unproven rather than inferred from non-periodontal models.

### 3.4 Maresins

Maresins (MaRs) were first identified in macrophages and include DHA-derived MaR1 and MaR2, together with n-3 DPA-derived maresin-like mediators (46, 47). Macrophage 12-LO converts DHA to a 13S, 14S-epoxide intermediate that is enzymatically hydrolyzed to MaR1 or MaR2; 15-LO is not the defining second step for MaR1 biosynthesis (108, 109). MaR1 stereoselectively activates LGR6 on phagocytes, enhancing phagocytosis and efferocytosis and engaging ERK/CREB-related pro-resolving signaling (50). MaR1 and MaR2 can limit PMN infiltration and favor pro-resolving/M2-like macrophage states (109). In periodontal and oral wound models, local MaR1 has accelerated healing, reduced alveolar bone resorption, increased CD206-positive macrophages, and promoted socket bone regeneration (86). These findings are encouraging but remain confined mainly to cell and rodent models.

MaR1 can regulate Treg/Th17 balance and inflammatory cytokine production in experimental systems (89). It also suppresses TLR4/MAPK/NF-κB signaling and can activate Nrf2-dependent antioxidant programs in non-periodontal inflammatory models (89, 90). Because excessive ROS and oxidative stress contribute to periodontitis, these mechanisms provide a plausible periodontal rationale (92), but plausibility is not equivalent to direct evidence. Periodontal-specific evidence for MaR1 is limited to a small number of *in vitro*, leukocyte, and animal wound/bone studies (86, 91, 99); evidence for MaR2 and n-3 DPA-derived maresins is even more limited. Findings from colitis, pulmonary, renal, vascular, and other inflammatory models should therefore be presented as mechanistic support requiring direct validation in periodontitis (**Supplementary Figure 2**).

## 4 Application of SPMs in periodontal therapy

### 4.1 Translational barriers and current clinical status

SPMs have therapeutic potential, but translation requires more than demonstrating biological activity. Native mediators often have short biological half-lives and are rapidly oxidized, dehydrogenated, or otherwise inactivated; reactive oxygen species and abundant metabolic enzymes in inflamed periodontal sites may further reduce local exposure (110–112). Additional barriers include stereochemically demanding and costly synthesis, limited large-scale manufacturing capacity, formulation compatibility, and sensitivity to oxygen, light, temperature, and storage time. No SPM formulation is currently approved specifically for periodontitis, and rigorous pharmacokinetic/pharmacodynamic studies, dose-ranging, safety assessment, stereochemical quality control, and storage-stability testing are needed. Metabolically stable analogues, including 15(R)-methyl-LXA4 and ATLa, can resist 15-hydroxyprostaglandin dehydrogenase and omega-oxidation, but analogue-specific receptor selectivity and toxicology still require confirmation (105–107). Direct human evidence is limited. A randomized phase-1 study of a BLXA4 oral rinse in 127 participants with gingival inflammation reported similar adverse-event frequencies and exploratory anti-inflammatory signals over 28 days, but it was short, early-phase, and not a definitive periodontitis efficacy trial (63). Trials of omega-3 fatty acids plus low-dose aspirin as adjuncts to debridement represent indirect pathway modulation rather than administration of a defined SPM (113). Thus, direct SPM treatment of periodontitis remains preclinical, and the human evidence should not be conflated with animal efficacy.

### 4.2 Advanced delivery and combination strategies

Local controlled delivery may protect SPMs from degradation, maintain therapeutic concentrations in the periodontal pocket, and reduce systemic exposure (110, 114, 115). Emerging platforms include smart or stimuli-responsive hydrogels, injectable biomaterials, lipid or polymeric nanocarriers, membrane-derived microparticles, and bioresponsive systems triggered by pH, reactive oxygen species, proteases, or bacterial products. In animal models, microparticle-delivered stable lipoxin analogues and hydrogel-based LXA4 delivery have reduced inflammatory infiltration and supported periodontal repair (55, 116, 117). Future devices should report loading efficiency, release kinetics, stereochemical integrity, periodontal retention, biodegradation, and batch stability rather than only endpoint efficacy. Clinically, SPMs are most plausibly developed as adjuncts to oral-hygiene instruction, scaling and root planing/subgingival instrumentation, or regenerative surgery with grafts and membranes—not as substitutes for biofilm disruption. Combination regimens may align microbial control with host-resolution and tissue-regeneration phases, but superiority over conventional care and optimal sequencing remain hypotheses that require factorial, controlled clinical trials. A recent preclinical study published in Dental Research provides a relevant design precedent for such temporally coordinated treatment. An engineered Escherichia coli Nissle 1917 platform combined anaerobically activated metronidazole delivery with externally triggered superoxide dismutase expression, thereby sequentially suppressing periodontal pathogens, reducing oxidative stress, and promoting periodontal ligament and alveolar bone repair in a rat model (118). Although this system did not administer an SPM, it illustrates how microbial control and subsequent modulation of the inflammatory microenvironment could be temporally integrated within a local periodontal intervention.

### 4.3 Precision periodontology and scalable production

A precision-periodontology framework could stratify patients by stage and grade, smoking and diabetes, baseline lipid-mediator balance (for example, SPM-to-LTB4/PGE2 profiles), receptor expression, neutrophil phenotype, and subgingival microbiome features (25, 30, 37). Such biomarkers could guide mediator selection, dose, and delivery interval, but none is yet validated for routine SPM prescribing. Supporting the biomarker and treatment-monitoring component of this framework, a longitudinal study published in Dental Research showed that salivary large extracellular vesicle-associated bacterial DNA differentiated periodontal health from Stage III/IV periodontitis. Signals associated with key periodontal pathogens, including *Porphyromonas gingivalis*, *Fusobacterium nucleatum*, and *Aggregatibacter actinomycetemcomitans*, also declined following non-surgical periodontal therapy (119). These findings provide an example of a non-invasive platform for monitoring periodontal status and treatment response, although its ability to guide SPM selection or dosing has not been validated. Evidence should therefore be separated by tier: periodontal cell and ex vivo studies demonstrate receptor-linked anti-inflammatory and regenerative mechanisms; animal studies show reduced leukocyte infiltration, osteoclast activity, dysbiosis, and alveolar bone loss; and direct clinical efficacy for periodontitis remains unestablished (11, 15, 17). For sustainable supply, microbial or recombinant lipoxygenase biocatalysis, enzyme and process engineering, semisynthesis, and metabolically stable analogues may reduce reliance on lengthy total synthesis (44, 45). However, scale-up must preserve absolute stereochemistry, purity, potency, and receptor selectivity. Translational programs should integrate manufacturing quality, formulation stability, PK/PD, biomarker validation, and clinically meaningful periodontal outcomes from the outset.

Key knowledge gaps remain. Animal models reproduce selected inflammatory and bone-loss features but do not fully capture the heterogeneity, chronicity, comorbidities, or long-term safety considerations of human periodontitis. Inter-individual SPM responses may vary with smoking, diabetes, baseline lipid-mediator profiles, receptor expression, and subgingival microbial ecology. No standardized periodontal dosing strategy has been established for mediator selection, concentration, delivery interval, treatment duration, or integration with debridement. Addressing these gaps will require biomarker-stratified dose-ranging trials with harmonized clinical and lipidomic endpoints.

## 5 Conclusion

Periodontitis can be viewed in part as a disease of failed inflammation resolution. Across SPM families, the most periodontally relevant functions are limitation of excessive neutrophil recruitment, enhancement of macrophage efferocytosis, attenuation of NF-κB/NLRP3 inflammatory amplification, and restraint of RANKL-driven osteoclastogenesis. These functions connect resolution to immune control and bone preservation, but they are not supported equally for every mediator or model. Resolvins and lipoxins have the most direct periodontal preclinical support, whereas protectin and maresin mechanisms are derived largely from non-periodontal models; receptor assignments also remain incomplete. Clinical translation is constrained by rapid metabolism, formulation and storage instability, costly stereoselective production, model-to-human differences, inter-patient response variability, and the absence of standardized dosing or an approved periodontal SPM product. Early human lipoxin-analogue and indirect omega-3/aspirin studies are encouraging but do not establish direct efficacy in periodontitis. Priority studies should combine validated PK/PD and safety endpoints with controlled local delivery, conventional debridement, biomarker-informed patient selection, and rigorous manufacturing quality. Such evidence is required before SPM-based adjuncts can be considered reproducible precision host-modulatory periodontal therapies.

## Supplementary material

Supplementary Figure 1 Initiation of periodontitis. *Porphyromonas gingivalis* (*P. gingivalis*) stimulates inflammatory mediator and reactive oxygen species production, contributing to matrix degradation and oxidative stress. Neutrophils and monocytes/macrophages migrate to inflamed sites to phagocytose bacteri. During resolution, these leukocytes produce SPMs that support microbial clearance, limit continued inflammatory recruitment, and create a tissue environment permissive for epithelial, connective-tissue, and bone repair.

Supplementary Figure 2 Proposed mechanisms by which SPMs may modulate periodontal inflammation. Dental plaque promotes neutrophil accumulation, inflammatory mediator release, an increased RANKL/OPG ratio, osteoclastogenesis, and tissue damage (left). Specialized pro-resolving mediators (SPMs) reduce neutrophil infiltration, promote M1-to-M2 macrophage polarization, efferocytosis, bacterial clearance, and Treg/Th17 balance (right). They also enhance periodontal ligament stem-cell proliferation and osteogenic differentiation, fibroblast-mediated repair, and osteoblast function, thereby restoring homeostasis and supporting bone regeneration.
